# Submucosal Injection Solution for Endoscopic Resection in Gastrointestinal Tract: A Traditional and Network Meta-Analysis

**DOI:** 10.1155/2015/702768

**Published:** 2015-01-29

**Authors:** Zhang Yu Huai, Wei Feng Xian, Luo Chang Jiang, Wang Xi Chen

**Affiliations:** Department of General Surgery, Lanzhou University Second Hospital, Lanzhou 730030, China

## Abstract

*Objective*. To explore and define the current optimal submucosal injection solution used in ESD and EMR for gastrointestinal tract neoplasms in terms of clinical outcomes and other aspects. *Methods*. PubMed, Cochrane Library, Embase, and clinical trials register center were searched with terms of “endoscopic resection” and “submucosal injection solution” to identify relevant randomized controlled trials (RCTs). Both direct comparison using traditional meta-analysis method and indirect comparison using network meta-analysis method were performed. *Results*. A total of 11 RCTs with 1152 patients were included. Meta-analysis showed that, compared with normal saline, other submucosal injection solutions induced a significant increase in terms of *en bloc* resection rate (*I* ^2^ = 0%, OR = 2.11, 95% CI (1.36, 3.26), and *P* = 0.008) and complete resection rate (*I* ^2^ = 0%, OR = 2.14, 95% CI (1.41, 3.24), and *P* = 0.0003); and there was no significant difference in the incidence of total complications (*I* ^2^ = 0%, OR = 0.87, 95% CI (0.59, 1.29), and *P* = 0.49). *Conclusions*. Other newly developed submucosal injection solutions significantly increased *en bloc* resection rate and complete resection rate and decreased bleeding rate and finical cost of endoscopic resection in gastrointestinal tract, while current evidence did not find the difference between them, which need to be explored by further studies.

## 1. Introduction

With the diagnosis technique and accuracy of early gastrointestinal (GI) tract cancer increased, endoscopic treatment is widely applied as a radical curative therapy [1]. Among kinds of operations, endoscopic mucosal resection (EMR) and endoscopic submucosal dissection (ESD) are the most frequently used as they are minimally invasive especially for sessile and flat polyps [2]. They are proposed as replacements for invasive surgery in early gastrointestinal neoplasm (with low risk of lymph-node metastasis) due to simplified procedure, decreased cost, improved quality of life, and favorable long-term benefit [3, 4].

ESD overcomes the limitation that when a lesion is larger than 20 mm, EMR could hardly enable* en bloc* resection, while compared with EMR it increases the operation time and the risk of complications such as bleeding and perforation [5]. In order to facilitate easier and safe performance of the two procedures, a submucosal injection solution is always required to lift the lesion from muscular layer. An optimal injection solution must primarily contribute to achievement of better clinical outcomes and meanwhile should firstly achieve and maintain the necessary submucosal lifting height and duration [6], secondly not influence the histological evaluation, thirdly not have tissue toxicity [7], and fourthly be easily prepared and administrated with low cost. Currently, various submucosal injection solutions including normal saline (NS), fibrinogen mixture (FM), dextrose (DE), glycerol, sodium hyaluronic acid (SHA), succinylated gelatin (SG), hydroxyethyl starch (HES), and mesna (ME) are developed. And many studies have compared the various injections in EMR and ESD for GI tract neoplasm in terms of clinical efficacy and safety.

However, the results of these studies are not completely consistent, and the number of direct comparison studies is not sufficient. So, in order to explore and define the current optimal submucosal injection solution used in ESD and EMR for GI tract neoplasm in terms of clinical outcomes, we performed this meta-analysis of all eligible randomized controlled trials.

## 2. Materials and Methods

### 2.1. Literature Search

A systematic search was performed on databases including PubMed (1966.01–2014.07), Cochrane Library (2014 Issue 07), Embase (1974.01–2014.07), and clinical trials register center (up to 2014.07). Search terms were (“endoscopic submucosal dissection” OR “ESD” OR “endoscopic mucosal resection” OR “EMR” OR “endoscopic resection”) AND (“submucosal injection solution” OR “hyaluronic acid” OR “normal Saline” OR “dextrose”). Medical subject headings and extend function were also used to identify all relevant studies including abstracts, comments, reviews, clinical studies, and citations.

### 2.2. Inclusion and Exclusion Criteria

Only randomized controlled trials (RCTs) published in English were eligible. Patients with gastrointestinal tract lesions who were willing to receive polypectomy were participants, and they were randomized to treatment group or control group. All the interventions were comparable between the two groups except for the submucosal injection solution used. The main outcome measures to evaluate therapeutic efficacy were* en bloc* resection (defined as resection without piecemeal separation) rate and complete resection (defined as absence of neoplastic tissue in the edge of the cut lesion) rate. Secondary outcome measures were the incidence of total complications, bleeding, perforation, and recurrence. The primary literature search results were scanned by abstracts and further assessment was reading the full-text by two reviewers independently.

### 2.3. Data Extraction and Quality Assessment

Two reviewers extracted basic information (such as the first author, publication year, group, and case) and data (main and secondary outcome measures) from the included studies. Methodological quality was assessed according to the method recommended by the Cochrane Handbook [8], mainly based on six items: randomization, allocation concealment, blinding, comparative baseline, >80% follow-up, and freedom of selective reporting. According to quality assessment results, studies were judged as levels A (all the six items were appropriate), B (four or five items were appropriate), and C (less than four items were appropriate). And any disagreements about eligibility, data, and quality assessment were resolved through discussion.

### 2.4. Statistical Analysis

We entered all the extracted data into a predesigned table and did traditional meta-analysis using Revman software (version 5.29, recommended by the Cochrane Collaboration). Chi-square and *I* ^2^ statistic were adopted to assess the heterogeneity between trials. Subgroup analysis was performed to explore the specific clinical outcomes and complications, and funnel plot was used to evaluate the potential risk of publication bias. Then pooled odds ratios (OR) and their 95% confidence intervals (95% CI) were summarized and used for network meta-analysis. We did network meta-analysis using ITC software (version 1.0, Canadian Agency for Drugs and Technologies in Health, Indirect Treatment Comparison Software, Ottawa, Ontario, Canada) [9]. The head-to-head indirect comparison was handled and then assigned a result in terms of statistical superiority/inferiority or no difference between the groups, and relevant forest plots were also presented.

## 3. Results

### 3.1. Flow Diagram of Trial Selection

**Figure 1:**
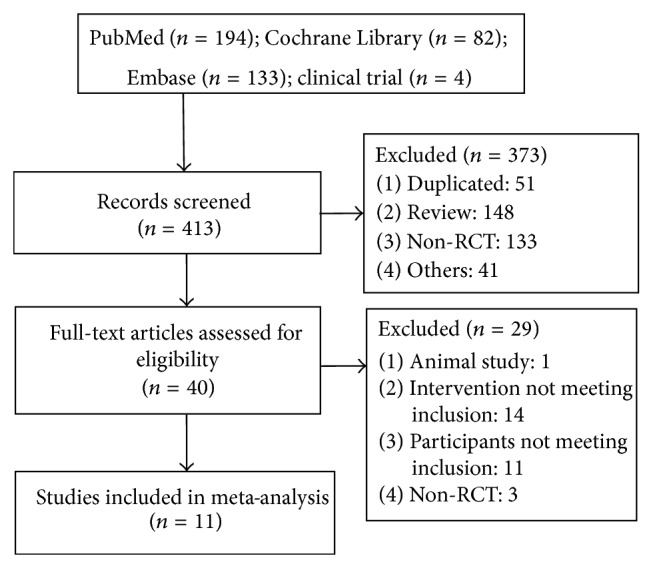
Flow diagram of trial selection.

In total, eleven RCTs [10–20] containing 1152 patients were included.  Figure 1 shows a flow diagram of trial selection from the initial search result to the final inclusion. The basic information about the publications, the participants, and the lesions was described in Table 1. The methodological method assessment showed that 7 RCTs reached a level of A [10–12, 15, 16, 19, 20], 2 RCTs reached a level of B [13, 17], and 2 RCTs reached a level of C [14, 18] (Table 2).

### 3.2. Direct Comparison of Submucosal Injection Solution with NS

#### 3.2.1. Pooled Analysis

Compared with normal saline (NS), other submucosal injection solutions used in ESD or EMR for gastrointestinal lesions induced a significant increase in terms of* en bloc* resection rate (*I* ^2^ = 0%, OR = 2.11, 95% CI (1.36, 3.26), and *P* = 0.008, Figure 3) and complete resection rate (*I* ^2^ = 0%, OR = 2.14, 95% CI (1.41, 3.24), and *P* = 0.0003, Figure 4). Though there was no significant difference in the incidence of total complications (*I* ^2^ = 0%, OR = 0.87, 95% CI (0.59, 1.29), and *P* = 0.49, Figure 5).

#### 3.2.2. Subgroup Analysis

Subgroup analysis indicated that, compared with NS, other submucosal injection solutions significantly increased* en bloc* resection rate (*I* ^2^ = 0%, OR = 0.62, 95% CI (0.39, 0.98), and *P* = 0.04) and complete resection rate (*I* ^2^ = 0%, OR = 0.62, 95% CI (0.39, 0.98), and *P* = 0.04) in EMR subgroup, as shown in Figure 3. Compared with NS, other submucosal injections also significantly increased* en bloc* resection rate (*I* ^2^ = 0%, OR= 0.62, 95% CI (0.39, 0.98), and *P* = 0.04) and complete resection rate (*I* ^2^ = 0%, OR = 0.62, 95% CI (0.39, 0.98), and *P* = 0.04) in ESD subgroup, as shown in Figure 4.

Subgroup analysis of complications indicated that, compared with NS, other submucosal injections significantly decreased the incidence of bleeding (*I* ^2^ = 0%, OR = 0.62, 95% CI (0.39, 0.98), and *P* = 0.04), while significantly increasing the incidence of postpolypectomy syndrome (*I* ^2^ = 0%, OR = 6.42, 95% CI (1.10, 37.62), and *P* = 0.04), and there was no significant difference in terms of perforation (*I* ^2^ = 0%, OR = 0.96, 95% CI (0.19, 4.87), and *P* = 0.96) and recurrence (*I* ^2^ = 0%, OR = 0.56, 95% CI (0.23, 1.38), and *P* = 0.21).

### 3.3. Indirect Comparison of Submucosal Injection Solutions

**Figure 2:**
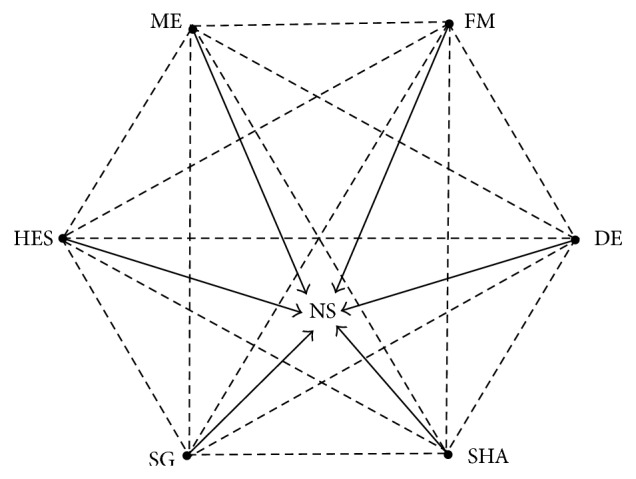
Network of clinical trials according to the comparison of specific classes of initial injected solutions. NS, normal saline; FM, fibrinogen mixture; DE, dextrose; SHA, sodium hyaluronic acid; SG, succinylated gelatin; HES, hydroxyethyl starch; and ME, mesna.

**Figure 3:**
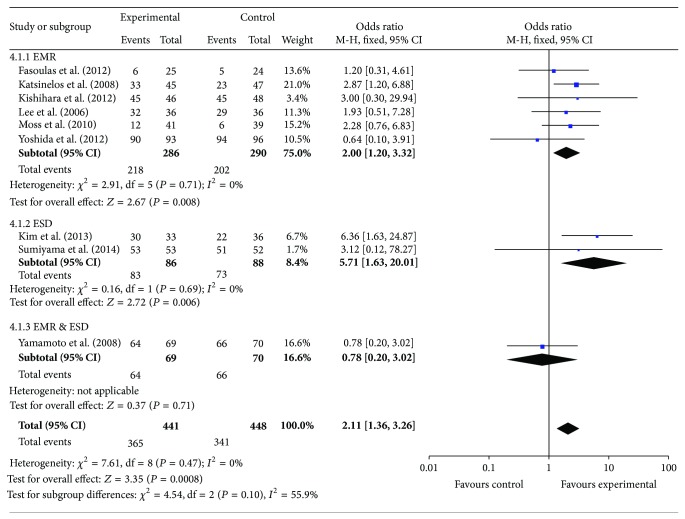
Meta-analysis results of* en bloc* resection rate between the two groups.

**Figure 4:**
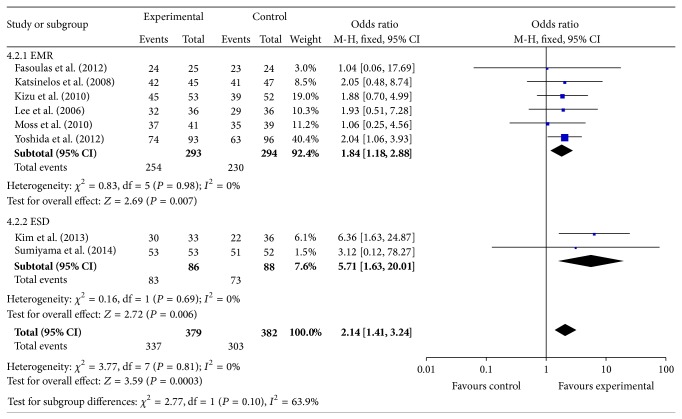
Meta-analysis results of complete resection rate between the two groups.

**Figure 5:**
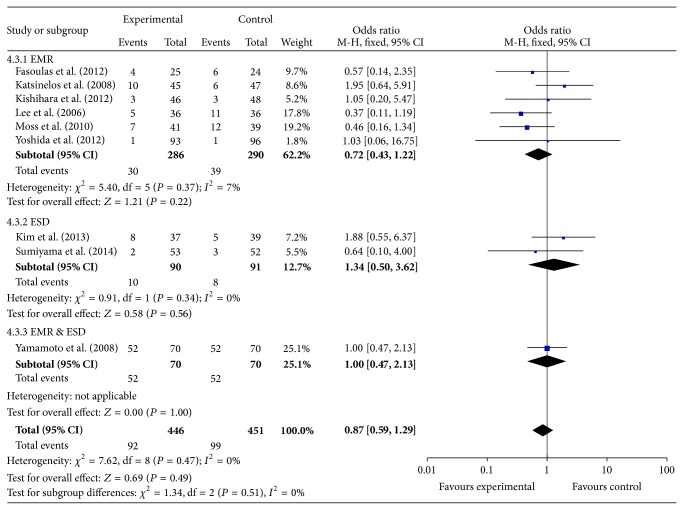
Subgroup meta-analysis of total complications.

Figure 2 shows the network of clinical trials according to the specific classes of initial injected solutions. Indirect comparison using network meta-analysis method according to the solution classes was mainly conducted in* en bloc* resection rate and complete resection rate, as shown in Table 3.

#### 3.3.1. *En Bloc* Resection Rate

The OR value for fibrinogen ranged from 0.18 to 0.43 compared with the others, and there was a significant difference between fibrinogen and dextrose (OR = 0.18, 95% CI (0.04, 0.89)). The OR value for dextrose ranged from 1.26 to 2.39 compared with the others, while the difference failed to reach any statistical significance. The OR value for hyaluronate acid ranged from 0.85 to 1.62 compared with the others, and there was no significant difference. The difference between succinylated gelatin and hydroxyethyl starch also failed to reach statistical significance.

#### 3.3.2. Complete Resection Rate

The OR value for fibrinogen ranged from 0.48 to 1.44 compared with the others, and there was no significant difference. The OR value for dextrose ranged from 0.66 to 1.97 compared with the others, and the difference failed to reach statistical significance. The OR value for hyaluronate acid ranged from 0.77 to 2.31 compared with the others, and there was no significant difference. The differences among succinylated gelatin, hydroxyethyl starch, and mesna also failed to reach statistical significance.

### 3.4. Publication Bias

**Figure 6:**
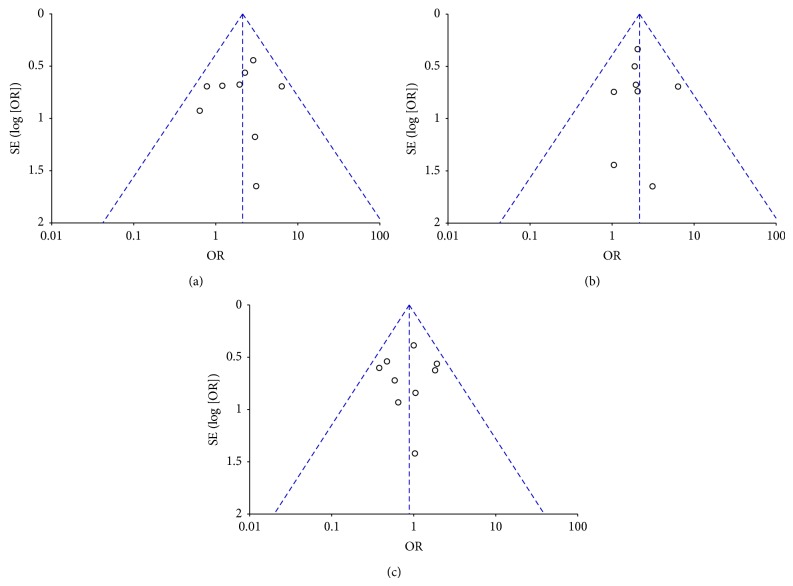
Funnel plot of publication bias. (a)* En bloc* resection rate, (b) complete resection rate, and (c) total complications.

Funnel plots were adopted to evaluate the publication bias. The shapes of funnel plots for* en bloc* resection rate, complete resection rate, and total complications did not reveal asymmetry, indicating no evidence of publication bias (Figure 6).

## 4. Discussion

This meta-analysis was the first to explore the influence of different classes of submucosal injection solutions for endoscopic resection and found a distinct advantage of other submucosal injection solutions than NS. The* en bloc* resection rate (82% versus 77%) and complete resection rate (89% versus 79%) were both significantly higher in the other submucosal injection solution group. Though there was no significant difference in the incidence of total complications, subgroup analysis indicated that other injection solutions significantly decreased the incidence of bleeding, while some of them increased the incidence of postpolypectomy syndrome. There was no significant difference between the two groups in terms of recurrence and perforation. Heterogeneity test and inverted funnel plot demonstrated that there were little heterogeneity and publication bias, and all the results were pooled in the fixed-effect models.

As known, EMR was mostly adopted for lesion size smaller than 20 mm, and ESD was mostly applied for lesion size larger than 20–30 mm with increased technical difficulties and equipment requirement [21]. Of the included studies, 6 applied EMR, 4 applied ESD, and 1 applied ESD and EMR according the lesion size. A previous meta-analysis demonstrated that there was no significant difference between ESD and EMR in terms of* en bloc* resection rate and complete resection rate, and ESD was only associated with prolonged operation time and increased complication incidence [22]. Therefore, the different method used in the trials would not influence the main outcome measure in the study. When the lesion size was large, it also had high possibilities to be diagnosed as carcinoma and invade into deep tissue [23, 24]; thus the difficulty of operation was really increased. Except one study reporting the average lesion size was larger than 20 mm, other studies all included patients with average lesion size smaller than 20 mm, and this difference may induce a lower* en bloc* resection rate and complete resection rate, which may be a source of heterogeneity. Meanwhile, lesion location may also have had some impacts on the results [10, 24], while we did not find any obvious difference between the studies as shown in Table 1.

When a lesion size is smaller than 20 mm, the required time for operation is short; although NS with low molecular weight is easy to diffuse to surrounding tissues, it can maintain enough mucosal elevation height and duration for operation [16], so this may explain the little difference between the other injection solutions and NS in terms of clinical outcomes for EMR. However, for lesion size larger than 20–30 mm, the required time is relatively long. Endoscopic operator sometimes needs to administer an additional injection when NS was used as submucosal injection solution; in this case the other injection solutions also had advantages in terms of operation time and additional injection [15], and all these terms are important for not only difficult operations but also nonexpert endoscopic operators.

So compared with NS, other newly developed submucosal injection solutions really improved the clinical outcomes. Because the pooled analysis results only reflected an overall effect of the other injection solutions, we further defined the separate effect of each injection solution to explore the more effective ones though network meta-analysis method. According to the type of injection solution, we divided all of them into 6 subgroups including fibrinogen mixture (FM), dextrose (DE), sodium hyaluronic acid (SHA), succinylated gelatin (SG), hydroxyethyl starch (HES), and mesna (ME). In aspect of* en bloc* resection, dextrose was significantly better than FM, and the other solutions also seemed to be better than FM, while all of them failed to reach statistical significance. And it seemed that dextrose also achieved a high* en bloc* resection rate compared to SHA, SG, and HES, while no significant difference was found. In aspect of complete resection, SHA and dextrose were both better than the others except for mesna, while it should be suspected that the result of mesna always had a wide 95% CI. Therefore, current evidence indicated that there was no significant difference between the newly developed injection solutions, and in aspects of* en bloc* resection rate and complete rate they did not significantly differ from each other. Meanwhile, the abilities of the other solutions to maintain the mucosal elevation seemed to be equivalent as they all declared that no additional injection was needed.

To precisely make a histologic diagnosis, a submucosal injection solution should not damage the cut tissue, and it was reported that hypertonic saline and dextrose caused immediate and delayed porcine tissue damage [7]. In contrast, NS, glycerol, SHA, and FM did not cause any apparent tissue damage, while no relevant studies were performed for SG, HES, and mesna [10]. Except for the fact that dextrose was reported to cause postpolypectomy syndrome more than NS (13% versus 2%), it was explained that the dextrose did not cause this itself but was injected too deeply into the muscular colonic wall and obliterated the local vascular supply [12]; FM had a high viscosity of fibrinogen which might conjecture the injection materials [10]; and SHA can to some extent stimulate the growth of residual tumor cells [25]. So SHA is a really ideal injection solution, except for its major disadvantages including high cost, no wide availability, and special storage requirement [26]. The reported price of SHA was $50–120/mL, FM was $0.2/mL, glycerol was $0.01–0.03/mL, SG was $0.02/mL, and NS was <$0.01/mL, while the price of HES and mesna was not reported. It was obvious that, compared with SHA, the other injection solutions all significantly reduced finical costs and were easily prepared.

There are still some possible limitations in the current study: (1) although the overall methodological quality is good, two studies have a poor quality with level of C [14, 18]; (2) as the relative small cases in the analysis, the results of FM and HES should be supported by more studies; (3) endoscopic operator was reported by most studies with performance experience of ESD and EMR > 300 cases, excepted one study that had unexperienced endoscopist [18]; and endoscopic operator's experience and learning curve really significantly influence the outcomes; the current study could not draw a conclusion on this; (4) glycerol was also a promising submucosal injection solution, while the current study could not quantitively analyze it because no relevant RCT was found.

In conclusion, other newly developed submucosal injection solutions significantly increased the* en bloc* resection rate and complete resection rate and decreased the bleeding rate and finical cost of endoscopic resection in GI tract, while current evidence did not find the difference between them, which need to be explored by further studies.

## Figures and Tables

**Table 1:** Characteristics of participants.

Study	Group	Case	Age	Sex	Size	Site	Method	Type
				(M/F)	(mm)			
Lee et al., 2006 [10]	Fibrinogen	36	61.4 ± 11.5	27/9	19.2 ± 6.3	Gastric, proximal 2/3 (11), distal 1/3 (25)	EMR	Adenoma (24), adenocarcinoma (12)
	NS	36	59.7 ± 11.2	21/15	16.8 ± 6.1	Gastric, proximal 2/3 (12), distal 1/3 (24)		Adenoma (25), adenocarcinoma (11)

Hurlstone et al., 2008 [11]	Dextrose	82	56 (29–84)^*^	42/40	18 (6–35)^*^	Colon, left (33), right (49)	EMR	LGD (58), HGD (19), carcinoma (5)
	0.4% SHA	81	58 (32–83)^*^	39/42	20 (4–40)^*^	Colon, left (36), right (45)		LGD (59), HGD (18), carcinoma (4)

Katsinelos et al., 2008 [12]	Dextrose	45	65 (42–82)^*^	20/25	>10	Rectosigmoid, rectum (27), sigmoid (18)	EMR	LGD (16), MGD (18), HGD (9), carcinoma (2)
	NS	47	69 (41–92)^*^	27/20		Rectosigmoid, rectum (27), sigmoid (20)		LGD (23), MGD (13), HGD (10), carcinoma (1)

Yamamoto et al., 2008 [13]	0.4% SHA	69	65.3 ± 8.1	57/12	5–20^*^	Gastric, proximal (8), body (30), distal (31)	ESD & EMR	Adenoma (18), adenocarcinoma (58)
	NS	70	66.1 ± 8.4	51/19		Gastric, proximal (8), body (28), distal (34)		Adenoma (17), adenocarcinoma (53)

Kizu et al., 2010 [14]	0.4% SHA	53	—	—	<20	Gastric	ESD	—
	NS	52						

Moss et al., 2010 [15]	SG	41	69 (64–76)^#^	22/19	40 (25–45)^#^	Colon, proximal to hepatic flexure (17)	EMR	Tubular (18), tubulovillous (16), sessile serrated adenoma (7)
	NS	39	67 (62–78)^#^	23/16	35 (30–50)^#^	Colon, proximal to hepatic flexure (14)		Tubular (11), tubulovillous (22), sessile serrated adenoma (6)

Fasoulas et al., 2012 [16]	HES	25	68 (43–82)^*^	16/9	4.5 (3.2–7)^*^	Colorectal, rectum (19), ascending colon (3), cecum (3)	EMR	Paris classification, O-II a (19), O-II b (6)
	NS	24	67 (48–88)^*^	8/16	4.6 (3.3–7.2)^*^	Colorectal, rectum (18), ascending colon (3), cecum (3)		Paris classification, O-II a (21), O-II b (3)

Kishihara et al., 2012 [17]	0.2% SHA	46	61.0 ± 9.0	21/25	11.3 ± 3.0	Colorectal, proximal (20), distal (22), rectum (4)	EMR	Adenoma (39), adenocarcinoma (7)
	NS	48	65.0 ± 8.0	32/16	12.5 ± 4.0	Colorectal, proximal (26), distal (16), rectum (6)		Adenoma (40), adenocarcinoma (8)

Yoshida et al., 2012 [18]	0.13% SHA	93	66 (23–85)^*^	62/31	8.9 (8–16)^*^	Colorectal, cecum to descending colon (49), rectum to sigmoid (44)	EMR	Adenoma (84), adenocarcinoma (9)
	NS	96	67 (35–89)^*^	63/33	8.2 (5–15)^*^	Colorectal, cecum to descending colon (50), rectum to sigmoid (46)		Adenoma (92), adenocarcinoma (4)

Kim et al., 2013 [19]	0.4% SHA	29	62.6 ± 9.2	25/12	14.2 ± 5.47	Gastric, antrum (23), angle (3), body (11)	ESD	Adenoma (31), atypia (1), adenocarcinoma (5)
	NS	34	62.4 ± 9.9	26/13	13.5 ± 4.35	Gastric, antrum (29), angle (3), body (7)		Adenoma (31), atypia (1), adenocarcinoma (7)

Sumiyama et al., 2014 [20]	Mesna	53	—	41/9	19.5 ± 11.5	Gastric, upper (7), middle (25), lower (21)	ESD	Adenoma (7), adenocarcinoma (46)
	NS	53	—	41/9	17.1 ± 10.1	Gastric, upper (12), middle (20), lower (20)		Adenoma (5), adenocarcinoma (47)

E, epinephrine; HES, hydroxyethyl starch; SG, succinylated gelatin.

^*^Median (range), ^#^median (interquartile range).

LGD, low-grade dysplasia; MGD, moderate-grade dysplasia; HGD, high-grade dysplasia.

**Table 2:** Quality assessment of included randomized controlled trials.

Study	State	Randomization	Allocation concealment	Blinding	Comparable baseline	>80% follow-up	Freedom of selective reporting	Level
Lee et al., 2006 [10]	Korea	Y, central controlled randomization	Y	Y, double blind	Y	Y	Y	A

Hurlstone et al., 2008 [11]	UK	Y, random sequence	Y	Y, double blind	Y	Y	Y	A

Katsinelos et al., 2008 [12]	Greece	Y, random number	Y	Y, double blind	Y	Y	Y	A

Yamamoto et al., 2008 [13]	Japan	Y, center controlled randomization	Unclear	Unclear	Y	Y	Y	B

Kizu et al., 2010 [14]	—	M	Unclear	Unclear	Y	Y	Unclear	C

Moss et al., 2010 [15]	Australia	Y, random sequence	Y	Y, double blind	Y	Y	Y	A

Fasoulas et al., 2012 [16]	Greece	Y, block balance random sequence	Y	Y, double blind	Y	Y	Y	A

Kishihara et al., 2012 [17]	Japan	M	Y	Unclear	Y	Y	Y	B

Yoshida et al., 2012 [18]	Japan	M	Unclear	N	Y	Y	Y	C

Kim et al., 2013 [19]	Korea	Y, random sequence	Y	Y, double blind	Y	Y	Y	A

Sumiyama et al., 2014 [20]	Japan	Y, computer generated random sequence	Y	Y, double blind	Y	Y	Y	A

M, the method was mentioned, but there was not a detailed description; Y, the method was reported with detailed description; Unclear, no relevant information was found in the study.

**Table 3:** Network meta-analysis comparing different classes of injection solutions.

Interventions	*En bloc* resection		Complete resection	
	Odds ratios (95% CI)	GRADE	Odds ratios (95% CI)	GRADE
Fibrinogen mixture				
Dextrose	0.18 (0.04, 0.89)	High	0.73 (0.11, 4.96)	Moderate
Hyaluronic acid	0.27 (0.06, 1.21)	Moderate	0.63 (0.15, 2.60)	Moderate
Succinylated gelatin	0.23 (0.04, 1.27)	Moderate	1.41 (0.21, 9.63)	Moderate
Hydroxyethyl starch	0.43 (0.07, 2.84)	Moderate	1.44 (0.06, 31.99)	Low
Mesna	NA	NA	0.48 (0.02, 15.42)	Low
Dextrose				
Hyaluronic acid	1.47 (0.47, 4.59)	Moderate	0.85 (0.18, 3.96)	Moderate
Succinylated gelatin	1.26 (0.31, 5.11)	Moderate	1.93 (0.25, 15.08)	Low
Hydroxyethyl starch	2.39 (0.48, 11.94)	Low	1.97 (0.08, 47.96)	Very low
Mesna	NA	NA	0.66 (0.02, 22.72)	Low
Hyaluronic acid				
Succinylated gelatin	0.85 (0.23, 3.19)	Moderate	2.26 (0.49, 10.52)	Low
Hydroxyethyl starch	1.62 (0.35, 7.52)	Moderate	2.31 (0.13, 41.29)	Very low
Mesna	NA	NA	0.77 (0.03, 20.39)	Low
Succinylated gelatin				
Hydroxyethyl starch	1.90 (0.33, 10.81)	Low	1.02 (0.04, 24.72)	Low
Mesna	NA	NA	0.34 (0.01, 11.56)	Low
Hydroxyethyl starch				
Mesna	NA	NA	0.33 (0.00, 27.72)	Very low

GRADE: Grading of Recommendations, Assessment, Development, and Evaluation.
